# Annotated genome sequence of temperate *Arthrobacter globiformis* phage, Jamun

**DOI:** 10.1128/mra.01440-25

**Published:** 2026-02-26

**Authors:** John Horangic, Julia Wright, Ashna Siddiqui, Claire Bartley, Cynthia Brogan, Annie Katanga, Mel Buzzanga, Kristen Johnson, Kyle MacLea

**Affiliations:** 1 Biotechnology Program, University of New Hampshire, Manchester, New Hampshire, USA; 2 Biology Program, University of New Hampshire, Manchester, New Hampshire, USA; 3 Graduate Program in Biotechnology: Industrial and Biomedical Sciences, University of New Hampshire, Manchester, New Hampshire, USA; 4 Department of Life Sciences, University of New Hampshire, Manchester, New Hampshire, USA; Portland State University, Portland, Oregon, USA

**Keywords:** actinobacteria, bacteriophage genetics, genome analysis, soil microbiology

## Abstract

Jamun is a temperate actinobacteriophage from cluster AS infecting *Arthrobacter globiformis* NRRL B-2979. The genome length is 38,821 bp and contains 63 predicted protein-coding genes, of which 10 are reverse genes. It has a tail length of 120 nm and a capsid width of 50 nm (Fig. 1).

## ANNOUNCEMENT

To further our understanding of phage diversity, we isolated a cluster AS, sub-cluster AS1, actinobacteriophage infecting *Arthrobacter globiformis* NRRL B-2979. Jamun was discovered in a soil sample taken 2 inches below the surface in Bedford, NH, near a road at 17.8°C, located at GPS coordinates 42.941,631 N, 71.474,407 W and was isolated using a peptone yeast calcium (PYCa) broth, which was added directly to the soil sample. This mixture was filtered using a 0.22 µm filter and then mixed with *A. globiformis* and PYCa top agar before plating onto PYCa agar. The plaque isolated from this initial experiment was purified and amplified using the SEA-PHAGES protocols, resulting in a high-titer lysate (HTL) with a concentration of 9 * 10^9^ pfu/mL (1).

The Promega Wizard DNA Clean-Up Kit (A7280, Promega, Madison, WI, USA) was used to extract the DNA from the HTL (1). Libraries were prepared using the NEB Ultra II FS kit (E7805S, NEB, Ipswich, MA, USA) and were sequenced with an Illumina MiSeq instrument, yielding 269,715 single-end 150 bp reads. Raw reads were trimmed/quality controlled and assembled into a single contig using Newbler v2.9 with default parameters (2). Sequencing coverage and genome completion were confirmed using Consed v29 (3). Genome termini were also determined using Consed v29 as described in (4). This genome has 38,821 bp with a GC content of 67.8% and was sequenced to a depth of ~1,042-fold.

Jamun was manually annotated with DNAMaster V5.23.6 (https://phagesdb.org/DNAMaster). Auto-annotated start sites from Glimmer V3.02 (5) and GeneMark V2.5 (6) were analyzed with data from Starterator V1.2 (7) and Phamerator actino_draft version v509 (8) to designate the location of start sites of genes. Gene function was determined by comparing these genes through BLASTp on NCBI V2.13 (9) and HHPred (https://toolkit.tuebingen.mpg.de/tools/hhpred). Genes were named following standards set by the SEA PHAGES program. No tRNA was found, as determined by the use of ARAGORN v1.2.38 (10) and tRNAscan-SE v2.0 (11). Default parameters for all software programs were used except where otherwise noted.

The bacteriophage Jamun has 63 annotated genes, with 38 genes having an assigned function. Similar to other AS1 phages, Jamun consists of structure and assembly genes in the left arm of the genome, and DNA metabolism genes in the right arm (12). In the middle of the genome, Jamun contains a predicted tyrosine integrase, immunity repressor, and excise (genes 33, 34, and 36) gene necessary for a temperate phage. The appearance of the plaques, clear with a turbid edge, also indicates Jamun is a temperate phage (13).

Phages Jamun and Chickaboom (GenBank accession no. PQ201088) possess similar genes. For example, their RecT-like ssDNA binding proteins are 93.64% identical (9). An average nucleotide identity (ANI) analysis, EZbiocloud (https://www.ezbiocloud.net/tools/ani, 14), identified Basilisk as the most similar phage, with an ANI of 92.58%.

**Fig 1:**
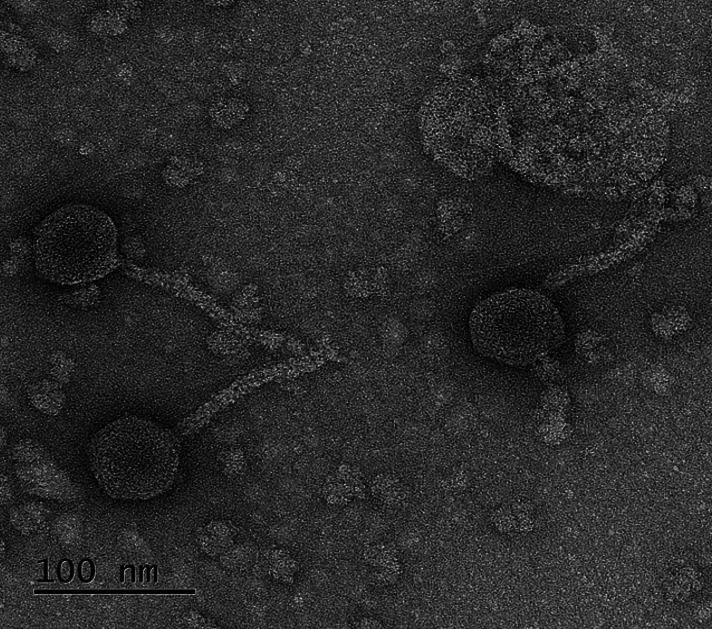
Jamun’s head is approximately 50 nm in diameter, and its tail is approximately 120 nm in length. The sample was prepared using a high-titer phage lysate deposited on a carbon conductive PELCO tab and stained with 1% uranyl acetate. This sample was then imaged using a Tecnai F20 microscope using an accelerating voltage of 200 kV at the Dartmouth Electron Microscope Facility, as described in Ulker et al. (15). (Scale bar = 100 nm).

## Data Availability

The genome sequence of bacteriophage Jamun has been deposited in DDBJ/ENA/GenBank under accession number OP297550. The raw Illumina data from BioSample SAMN26725081 were submitted to the NCBI Sequence Read Archive (SRA) under experiment accession number SRX14483241.
